# Effect of Three Different Liquid Medias in the Sorption and Solubility of Luting Cements: An In Vitro Study

**DOI:** 10.7759/cureus.47311

**Published:** 2023-10-19

**Authors:** Lakshmi Radhakrishnan, Nandakumar K, Amal Jassim, Aysha Mohamed Ali KP

**Affiliations:** 1 Department of Prosthodontics, The Muslim Educational Society (MES) Dental College, Perinthalmanna, IND

**Keywords:** fixed prosthesis, liquid medias, dimensional stability, solubility, sorption

## Abstract

Background

Among the various mechanical and biological properties of luting cement, the most important are its resistance to disintegration, degradation, and stability in the oral cavity. The sorption and solubility of cement alter the mechanical properties by impeding the half-life of the filling. It also leads to variations in dimensions, discoloration, and margin breakage. It is, therefore, essential to choose a low-solubility cement since there is always an interaction between teeth and restorative margins. The aim of this study is to assess and compare the solubility and sorption values of three different luting cements in three liquid media.

Materials and methods

Three luting cements were used for the investigation. Disc-shaped specimens of the cement, which were of 10 mm diameter and 2 mm height, were prepared. The sample included a total of 126 disc-shaped specimens made up of three materials, glass ionomer cement (GIC), resin cement, and resin-modified GIC, which were used in three liquid media (14 of each material in each medium). Fourteen specimens of each material were placed in glass vials containing 20 ml of each medium: distilled water, artificial saliva, and carbonated water. The samples were then put in an incubator at 37 °C. The measurements and masses of the samples were documented on days one, three, seven, 14, 21, 28, and 35. The samples were taken out of the solution after five weeks and stored in a desiccator with calcium sulphate for another five weeks. The weight and dimensional changes were estimated on days one, three, seven, 14, 21, 28, and 35. The values of water sorption (W_SO_) and solubility (W_SL_) were estimated. To determine the mean and standard deviation of each cohort, descriptive statistics were employed. Utilizing the Shapiro-Wilkinson test, the normality was determined. An independent test was used to determine the difference between all pairs of groups, while one-way ANOVA, Dunn test, and post hoc analysis were used to establish the distinction between the three groups.

Results

One-way ANOVA showed that significant differences existed among the groups: resin cement showed the least sorption and solubility, resin-modified GIC showed the highest solubility in distilled water (0.40 ± 0.03), and GIC showed the highest solubility in both artificial saliva (0.36 ± 0.03) and carbonated water (0.04 ± 0.05).

Conclusion

Considering the experimental outcomes and the limitations of an in vitro investigation, it was concluded that in the complex setting of the oral environment, this selection procedure is crucial for maintaining mechanical strength and for the long lifespan of dental restorations.

## Introduction

The structural integrity and dimensional stability of a fixed prosthesis (which are connected to the water sorption and solubility of luting cement) are crucial to the therapeutic effectiveness of a prosthesis [1]. Water consumption increases weight and solubility due to sorption, and the cement's mechanical properties, including flexural strength, rigidity, and mechanical stability, are negatively impacted by water sorption. This results in material components being washed away or dissolving and entering the oral cavity [2]. Moreover, dimensional fluctuation, loss of retention, material deterioration, and discoloration further lower the restoration's aesthetic standard [3]. With advancements in dental science, permanent cementation of metal-ceramic, all-ceramic restorations, porcelain veneers, and, more recently, laminate, inlay, and onlay restorations are increasingly being performed using dental cement consisting of a resin matrix, such as resin-modified glass ionomer cement (RMGIC), polyacid-modified composite resin cement (compomer), and self-etching, self-adhesive composite resin cement [4, 5].

The mechanical and physical qualities of resin luting cement are better than those of conventional luting cement [6]. The resin matrix of a polymer is where the majority of water absorption takes place. The absorbed water serves as a plasticizer, degrading the filler-matrix interface and causing color shifts and other aesthetic issues in the restoration [7]. Solubility also results in the formation of harmful by-products, including formaldehyde and methacrylic acid. These by-products, as well as any lingering monomers, fillers, or activators from the polymerization process, might accumulate and cause harm to the oral soft tissues [8].

Selecting an appropriate luting cement based on the specific clinical context and exposure to oral fluids can significantly impact the mechanical stability and longevity of dental restorations. This can aid clinicians in making informed decisions regarding the selection of luting cement for specific clinical situations, ultimately contributing to the long-term success of dental restorations. 

Therefore, the aim of this study is to compare the sorption and solubility of three distinct luting cements, namely, glass ionomer cement (GIC), resin-modified GIC, and resin cement in distilled water, artificial saliva, and carbonated water.

## Materials and methods

This in vitro study was carried out in the Department of Prosthodontics Crown and Bridge & Department of Biochemistry, the Muslim Educational Society (MES) Medical College, Perinthalmanna, Kerala, and was approved by the Institutional Ethical Committee (Approval No. IEC/MES/57/2020). For this study, disc-shaped cement samples of dimensions 10 mm diameter and 2 mm height were prepared. Powder and liquid were mixed in a specific ratio as per the manufacturer's guidelines. This mixture was then placed into a mold designed to produce discs with dimensions of 10 mm in diameter and 2 mm in height. The mold was subjected to a compressive force to ensure uniformity and to eliminate air bubbles. It was then allowed to be set in a controlled environment for the time recommended by the manufacturer. After setting, the RMGIC discs and resin discs were carefully removed from the mold and polished to ensure a smooth surface. The luting cements taken for this study were glass ionomer cement, resin cement, and RMGIC. The cements were mixed in consonance with the manufacturer's guidelines. The samples were given an appropriate period of time to set. A light-curing gun and curing units were used for RMGIC and resin cement to ensure total photoactivation and uniform curing. For sorption and solubility tests, the disc-shaped specimens were grounded by silicon carbide paper to get rid of unevenness.

The minimum sample size in each group was ($\mu$) = 40, and in this study, the sample size in each group was 42. This study comprised three groups; thus, a total of 126 (42×3) samples were used.

**Figure 1:**
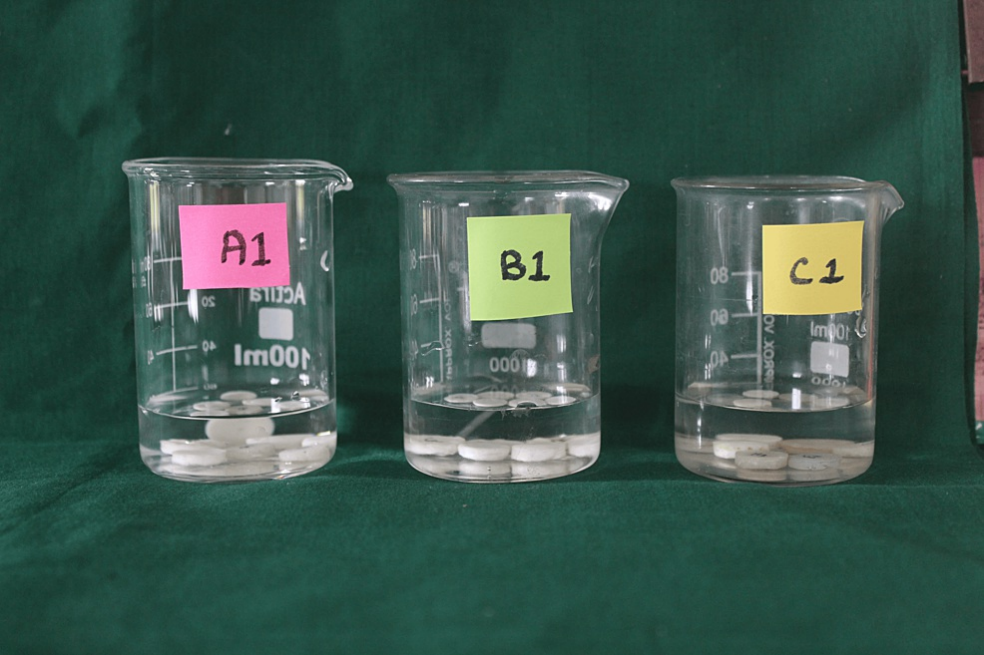
Discs in a medium of artificial saliva

According to the estimated sample size, the 126 samples were distributed equally in three categories: 42 cement discs in each medium of artificial saliva, distilled water, and carbonated water (Figure 1)

**Figure 2:**
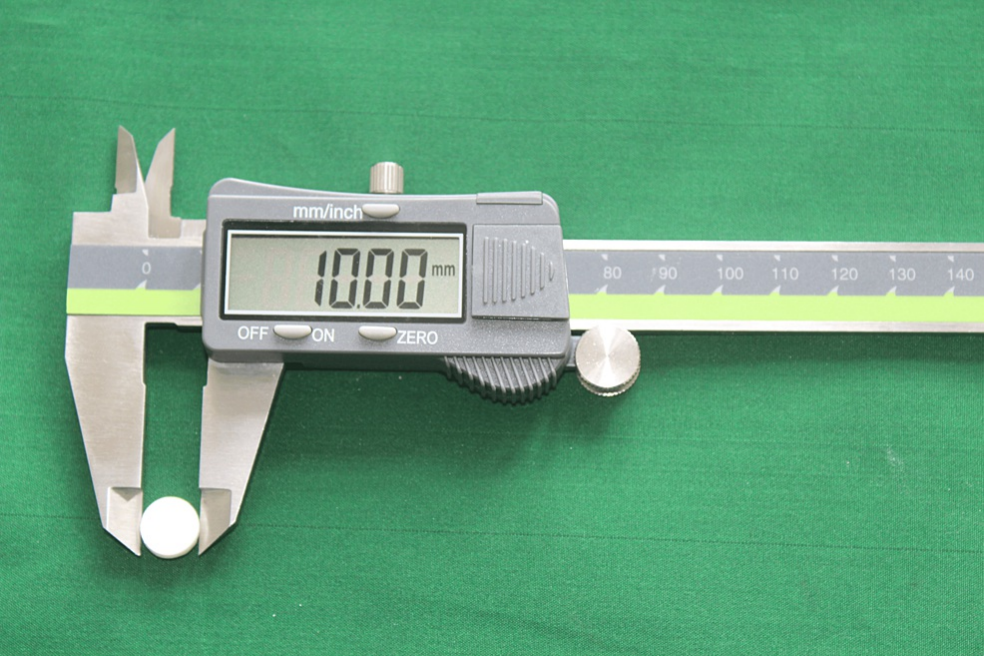
Measurements were done by vernier caliper

Other restorative cements such as zinc phosphate and zinc polycarboxylate and liquid media such as acids, ethanol, and mouth rinses were not considered in the study. The samples were weighed $𝑚$0 with an analytical scale. The diameter and thickness were measured with a digital caliper (Figure 2).

The volume was estimated in cubic millimeters: V=π × $𝑟$2 × h, where r was the mean sample radius, and h was the mean sample thickness. In each category, 14 samples were placed in three glass vials containing 20 ml each of distilled water, carbonated water, and artificial saliva. These vials were then covered with aluminum foil to prevent light exposure and kept at 37 °C in the incubator. The dimensions and the mass of samples were documented on days one and three and then at the end of the first, second, third, fourth, and fifth weeks. During every time frame, the samples were taken out and dried using a soft paper towel and were then weighed, measured, and placed back in the medium. The samples were then taken out of the solution after five weeks and stored in a desiccator with calcium sulfate for another five weeks. The mass and dimensional changes were measured on days one and three and then after the first, second, third, fourth, and fifth weeks.

The formulae used to determine sorption (W_SO_) and solubility (W_SL_) are as follows:

W_SO_ =m1-m2/V

W_SL_= m0-m2/V

Where m0 is the specimen mass before immersion (mg), m1 is the specimen after immersion, and m2 is the specimen after desiccation (mg).

Estimation of statistics was done using the statistical package SPSS 26.0 (IBM Inc., Armonk, New York), and the level of significance was set at p<0.05. To determine the mean and standard deviation of each cohort, descriptive statistics were employed. Utilizing the Shapiro-Wilkinson test, the normality was determined. An independent test was utilized to determine the comparison among all pairs of groups, while one-way ANOVA and, thereafter, Dunn test and post hoc analysis were used to establish the comparison between the three groups.

## Results

The comparison of sorption in GIC, resin, and resin-modified GIC groups was done using a two-tailed independent t-test (Table 1).

**Table 1:** Comparison of sorption within the groups **p≤0.001 was statistically highly significant; *p≤0.05 was statistically significant GIC - glass ionomer cement, RMGIC - resin-modified glass ionomer cement DW—distilled water, AS—artificial saliva, CW—carbonated water

Cements	Medium	Immersion SD±mean	Desiccation SD±mean	Independent t-test (p-value)	Kruskal Wallis test (p-value)	Dunn test (p-value)
GIC	Distilled water	0.29±0.03	0.26±0.03	0.0001**	0.001**	DW vs AS: 0.03*
	Artificial saliva	0.27±0.02	0.25±0.01	0.0003**	0.001**	DW vs CW: 0.42
	Carbonated water	0.30±0.02	0.28±0.03	0.0001**		AS vs CW: 0.001**
Resin cement	Distilled water	0.24±0.01	0.22±0.03	0.0001**	0.0001**	DW vs AS: 0.42
	Artificial saliva	0.25±0.02	0.23±0.01	0.0001**	0.0001**	DW vs CW: 0.0001**
	Carbonated water	0.20±0.03	0.19±0.03	0.0001**		AS vs CW: 0.0001**
RMGIC	Distilled water	0.25±0.03	0.23±0.03	0.0001**	0.04*	DW vs AS: 0.42
	Artificial saliva	0.26±0.03	0.24±0.03	0.0001**	0.03*	DW vs CW: 0.42
	Carbonated water	0.24±0.02	0.22±0.06	0.0001**		AS vs CW: 0.03*

The statistical significance was reported to be high with respect to all the test solutions (p≤0.001). The Kruskal Wallis test also showed a higher significance than RMGIC with a significance value of p≤0.05. Paired comparisons by the Dunn test showed the highest statistical significance in sorption in resin cement (except distilled water vs artificial saliva) and the least in RMGIC (only artificial saliva vs carbonated water).

The mean sorption comparison of three materials in three media was done using a two-tailed one-way ANOVA test (Table 2).

**Table 2:** Comparison of sorption between the groups **P≤0.001, statistically highly significant; *P≤0.05, statistically significant GIC - glass ionomer cement, RMGIC - resin-modified glass ionomer cement

Medium	Cements	Immersion SD±mean	Desiccation SD±mean	Kruskal-Wallis (p-value)	Dunn test (p-value)
Distilled water	GIC	0.29±0.03	0.26±0.03	0.0001**	GIC vs Resin: 0.0001**
	Resin Cement	0.24±0.04	0.22±0.03	0.0001**	Resin vs RMGIC: 0.001**
	RMGIC	0.25±0.03	0.23±0.03		RMGIC vs GIC: 0.04*
Artificial saliva	GIC	0.27±0.02	0.25±0.02	0.08	GIC vs Resin: 0.06
	Resin cement	0.25±0.04	0.23±0.04	0.09	Resin vs RMGIC: 0.34
	RMGIC	0.26±0.03	0.24±0.03		RMGIC vs GIC: 0.23
Carbonated water	GIC	0.30±0.02	0.28±0.02	0.0001**	GIC vs Resin: 0.0001**
	Resin cement	0.20±0.03	0.19±0.03	0.0001**	Resin vs RMGIC: 0.0001**
	RMGIC	0.24±0.07	0.22±0.06		RMGIC vs GIC: 0.0001**

Immersion and desiccation mean values were reported to have high statistical significance for distilled water and carbonated water, while artificial saliva showed comparatively the highest sorption in glass ionomer cement. However, the difference (immersion and desiccation mean values) was not statistically significant. For carbonated water, more sorption was seen in GIC, followed by resin-modified GIC and resin cement.

A comparison of solubility between groups was done, and the results showed that resin-modified GIC had the highest solubility in water, followed by GIC, and the least by resin cement. In addition, it was observed that GIC showed the highest solubility in artificial saliva and carbonated water, followed by resin-modified GIC and resin cement. It showed a high statistical significance in the Kruskal Wallis test (Table 3).

**Table 3:** Comparison of solubility. **p≤0.001, statistically highly significant; *p≤0.05, statistically significant GIC - glass ionomer cement, RMGIC - resin-modified glass ionomer cement DW—distilled water, AS—artificial saliva, CW—carbonated water

Medium	Cements	Immersion SD±mean	Post Hoc Test	Kruskal- Wallis (p- value)	Dunn test (p-value)
Distilled water	GIC	0.37±0.03	GIC vs resin: 0.06	0.0001**	GIC vs resin: 0.06
	Resin cement	0.35±0.03	GIC vs RMGIC: 0.0001**		GIC vs RMGIC: 0.0001**
	RMGIC	0.40±0.03	Resin vs RMGICL: 0.001*		Resin vs RMGICL: 0.001*
Artificial saliva	GIC	0.36±0.03	GIC vs resin: 0.0001**	0.0001**	GIC vs resin: 0.0001**
	Resin cement	0.31±0.02	GIC vs RMGIC: 0.0001**		GIC vs RMGIC: 0.05
	RMGIC	0.33±0.04	Resin vs RMGICL: 0.05		Resin vs RMGICL: 0.0001*
Carbonated water	GIC	0.34±0.05	GIC vs resin: 0.0001**	0.0001**	GIC vs resin: 0.0001**
	Resin cement	0.30±0.02	GIC vs RMGIC: 0.48		GIC vs RMGIC: 0.003*
	RMGIC	0.32±0.04	Resin vs RMGIC: 0.003*		Resin vs RMGIC: 0.0001*

## Discussion

Adsorption and absorption are the characteristics of sorption in which the former is a surface phenomenon, and the latter utilizes diffusion for transporting water particles inside a solid [9]. The solubility of a material is defined by the saturation concentration of a solution of that substance in another solvent. During testing, particles will be absent [10]. Routinely used luting cements were employed in this study to evaluate solubility and sorption [11].

Diffusion of water often follows one of two patterns: the free volumetric theory, wherein it occurs via a micro void with no link to the polar molecules, and the interaction theory, in which the material binds sequentially to hydrophilic groups [12].

A comparison of sorption within the GIC group was done, and the result showed that GIC sorption was the greatest in carbonated water and the least in artificial saliva. Yanikoglu et al. [13] discovered that a medium with pH 7 provided the greatest stability for cements. Artificial saliva taken for the research lacked enzymes such as anhydrase, amylase, lysozymes, and other esterases present in the oral cavity. This could explain why the solubility and sorption figures were so low for GIC in artificial saliva [14]. Moreover, due to the acidic bacteria in the mouth, surface disintegration may have occurred after prolonged exposure to these enzymes [15].

Likewise, sorption within resin cement in distilled water, artificial saliva, and carbonated water showed more sorption in artificial saliva and the least in carbonated water. McKenzie MA et al. [16] in their study suggested that cement's resistance to dissolving is composition-dependent rather than pH-dependent. The least sorption in carbonated water (pH 3.1) was due to phosphoric acid, which could chelate with calcium in the filling material and create insoluble complexes. When a material is subjected to acid, it opens up more readily to water molecules entering the polymer network, leading to more sorption [17].

Sorption within the resin-modified GIC group was the greatest in artificial saliva and the least in carbonated water. Hygroscopic expansion caused by the sorption of RMGIC could have exerted undesirable stresses on the tooth as well as the filling. A major monomer component, hydroxyethyl methacrylate (HEMA), is thought to be responsible for this and could be helpful in countering the polymerization shrinkage [18]. Knobloch et al. [19] and Leevailoj et al. [20], in their investigations, have demonstrated that RMGIC showed between 8% and 14% water sorption. With regard to these results, the usage of these for cementation of all-ceramic crowns appears controversial. Considering the findings of this investigation, it might seem that it is a deplorable choice.

A comparison of sorption in groups for distilled water showed more sorption in GIC followed by resin-modified GIC than in resin cement. Glass ionomer cements are shown to have limitations that include early vulnerability to moisture or water absorption. Key [21] reported in 2006 that the clinical qualities of GIC rely upon early defense against hydration, and dehydration is crucial since it is weakened by moisture while desiccation results in shrinkage and cracks. Conventional GIC dissolves and can be washed away from surfaces; it diffuses through voids or the bulk of cement [17]. The results of Yoruc and Karaaslan's work [22] in 2007 explained that water sorption was the greatest on the initial day of immersion, followed by progressive ingestion over the course duration until an equilibrium level was established, and absorption of GIC was mostly determined by material composition.

In this study, the resin cement exhibited the least sorption in all three media. The rate of sorption of resin cement depends on filler content; that is, low-filler resins have increased water sorption [23]. Kalachandra et al. [24] reported that the nature and quality of fillers and matrix links is an important factor.

In this investigation, GIC showed the highest solubility in artificial saliva and carbonated water, followed by resin-modified GIC and resin cement. These findings are consistent with those of research by Toledano et al. [25], in which they achieved water sorption and solubility values that were higher for RMGIC when compared with other resin-based materials; it could be due to filler content and HEMA. Mese et al. also [26] investigated the values of sorption and solubility of RMGIC in other materials and discovered them to be higher, which corresponds to the results of this study.

Resin cement showed the least solubility due to the presence of bis-GMA and inorganic filler particles; also, it was directly proportional. In addition, voids are incorporated while mixing the cement, thus leading to the formation of oxygen-inhibition zones of unpolymerized materials, which have an impact on the solubility of the set cement [27]. During conversion, many leachable components accumulate, leading to increased solubility [25].

The water absorbed by RMGIC's polymer matrix may cause filler matrix debonding or hydrolytic degradation of fillers, weakening the mechanical characteristics of the composite. Hydrolytic disintegration occurs when resin chemical bonds break or water softens, leading to unreacted monomers dissolving and leaching from resin samples in the water. For clinical relevance, special attention must be paid while cementing the post, as resin-modified cement leads to the fracture of the filling at the root part. The infiltration depends on the size and the time course. The time needed for a specimen to reach equilibrium with water decreases in proportion to its size, and water-absorbing materials are less stable in the short term [28].

Because of the use of diverse time frames and measuring units, comparing the results from different studies was challenging. In addition, the minute wear of the surface due to repeated handling could cause a specimen to lose weight [11]. After desiccation, the cement's final volume often decreased. It was thought that this was caused by the water replacing the unpolymerized and soluble components in the storage solutions. The use of advanced analytical balance equipment with higher resolution can help in obtaining more accurate measurements of weight changes. Employ microscopic analysis techniques, such as scanning electron microscopy (SEM) or atomic force microscopy (AFM), to examine the surface and structural changes in the luting cement specimens over time. These techniques can provide valuable insights into micro-level interactions. Using chemical analysis methods to detect and quantify specific ions or compounds released during solubility testing. This can help in understanding the chemical changes occurring within the luting cement. Extension of the duration of your study to assess sorption and solubility over a more extended period. Some materials may exhibit different behaviors over time, and longer-term studies can provide a more comprehensive understanding of their performance. Implementation of dynamic testing methods that simulate oral conditions more accurately. For instance, using a dynamic flow cell system to continuously circulate the testing medium over the specimens can better mimic the fluid dynamics in the oral cavity.

This study's limitations stem from its in vitro design, which does not precisely replicate the complex oral environment. While artificial saliva and carbonated water were used to simulate oral circumstances, the absence of biological components, including oral microbiota, enzyme activity, and salivary flow, may have had a different impact on the results than in real-world settings. Moreover, a greater sample size could produce more reliable and compelling results, lowering the possibility of statistical variations. This study, however, used a very small sample size of 14 specimens for each substance in each liquid medium. Further research that incorporates a more comprehensive representation of oral dynamics and considers a larger sample size over extended timeframes would contribute to a more comprehensive understanding of the clinical implications of luting cement solubility and sorption.

## Conclusions

In conclusion, this study has provided enhanced knowledge of the solubility and sorption characteristics of luting cement when exposed to various liquid media. The differences observed among the different materials highlight the need for selecting the most appropriate cement to maintain the mechanical stability and longevity of dental restorations in a complicated oral environment.
